# In-vitro and In-silico evaluation of antimicrobial and antibiofilm effect of Neem oil and Calcium hydroxide nanoparticles against *Mutans Streptococci* and *Enterococcus faecalis* isolated from endodontic infections

**DOI:** 10.1038/s41598-024-75669-7

**Published:** 2024-11-02

**Authors:** Wedad M. Nageeb, Sherouk Hussein Adam, Nasr Hashem, Nelly Abdelsalam

**Affiliations:** 1 Department of Medical Microbiology and Immunology, Faculty of Medicine, Suez Canal University, Ismailia, Egypt; 2 Department of Endodontics, Faculty of Dentistry, Suez Canal University, Ismailia, Egypt

**Keywords:** *Streptococcus mutans*, *Streptococcus sobrinus*, Neem antibacterial, Calcium hydroxide, Endodontic medications, Nanoparticles, Antibiofilm effect, Translational research, Applied microbiology

## Abstract

Different Streptococcal species including *Streptococcus mutans*, *Streptococcus sobrinus* and *Enterococcus faecalis* are commonly isolated in root canal infections including refractory, recurrent, and persistent cases. Calcium hydroxide (Ca (OH)_2_) has been widely used in endodontics as an intracanal medicament. However, using new antimicrobial herbal alternatives offers promising potentials which can be additionally enhanced by using nanoparticles (NPs). In this study, we evaluate the antimicrobial efficacy and antibiofilm effect of Neem oil including its NPs preparations and we compare the effect of conventional Ca (OH)_2_ to Ca (OH)_2_ NPs using standard disc diffusion method and quantitative microtitre dish biofilm formation assay against common pathogens isolated from root canal samples. Molecular docking was used to test the binding of 10 Streptococcal macromolecules to 5 candidate neem active constituents. Neem NPs 0.125 mg/ml showed better antibacterial effect than both Neem 15 mg/ml and Neem 0.15 mg/ml. Ca (OH)_2_ NPs 0.125 mg/ml also showed better antibacterial effect than each of Ca (OH)_2_ 10 mg/ml and Ca (OH)_2_ 0.1 mg/ml. Best biofilm mass inhibition was achieved by Neem oil 0.15 mg/ml at 74.55% ( IQ: 67.36–87.65) and Neem NPs 0.0125 mg/ml at 59.33% (IQ: 51-–75.27). For Ca (OH)_2_, the best biofilm mass inhibition was observed with Ca (OH)_2_ NPs 0.125 mg/ml at 54.7% (IQ: 42.37– 77.25). Both neem oil and neem NPs show promising antibacterial and antibiofilm potential against *Mutans Streptococci* group at low concentrations and hence are good candidates for use as endodontic medications. In silico analysis shows that both Sitosterol and Gedunin appear to be important active constituents of neem and possible drug candidates. Additionally, Ca (OH)_2_ NPs showed significantly higher antimicrobial effect against *Mutans streptococci* group than conventional Ca (OH)_2_ preparations.

## Introduction

Caries is well recognized as the primary factor responsible for the exposure of tooth pulp, that subsequently leads to the development of more progressive forms of infection. The development of dental caries follows a sequential cascade of reactions starting with pulpal disease, then progressing to pulpitis, pulpal necrosis, and periapical disease (apical periodontitis)^1^. Additionally, the infection can disseminate from the root canal to the adjacent tissues, leading to cellulitis. It is well documented that endodontic apical periodontitis is caused and maintained by microorganisms and their harmful metabolic products. Primary and secondary endodontic infections have been clearly linked to the presence of Streptococcal species. Virulence of *Streptococcus mutans* has been linked to its ability to adhere to collagen and to form biofilms. The organism has been considered a major pathogen that underlies caries and periodontal disease development^2^. *S. mutans* is frequently isolated from endodontic infections, including root canals of symptomatic and asymptomatic necrotic teeth, in addition to its prominent role in acute apical abscesses^3^. Different Streptococcal species, including *Streptococcus mutans*, *Streptococcus sobrinus,* and *Enterococcus faecalis,* are commonly isolated in root canal infections, including refractory and recurrent apical periodontitis and persistent cases^4^. The study of *S. mutans* isolated from the oral cavity is of great interest because of its key function in caries development as well as its relationship with extra-oral illnesses such as infective endocarditis (IE)^5^.

Apical inflammatory lesions are primarily caused by microorganisms and the primary aim of endodontic treatment is to prevent pulpal and peri radicular infections. A medicament in endodontics is defined as an antimicrobial substance placed inside the root canal between treatment sessions with the aim of destroying any remaining microorganisms and preventing reinfection^6^. A commonly and frequently used intracanal medicament after chemo-mechanical preparation is calcium hydroxide (Ca (OH)_2_). The release of hydroxyl ions when Ca (OH)_2_ comes in contact with aqueous fluids is responsible for its antimicrobial effect through creating a highly alkaline environment where most pathogens cannot survive^7^. Protein denaturation, DNA damage, and damage to the bacterial membrane are the most likely causes of its bacterial killing effect. Despite possessing some disadvantages, Ca (OH)_2_ is currently the preferred material for intracanal medication and root canal dressings.

However, research has shown that extended exposure to Ca (OH)_2_ lowers dentine’s flexural strength and fracture resistance. Furthermore, the movement of hydroxyl ions into acidic root resorption zones enhances the development of inflammatory root resorption^6^. Moreover, standard methods cannot completely remove the Ca (OH)_2_ paste from the root canal walls. Ca (OH)_2_ paste is well tolerated by bone and dental pulp tissues, although its impact on periodontal tissue is debatable. Some studies have reported a detrimental effect on periodontal tissues that could negatively influence marginal soft tissue healing (Hauman & Love, 2003). Calcium hydroxide has been used as intracanal medicaments in dentistry for several decades, however, its spectrum of antimicrobial effect is controversial, carrying some limitations^8^. This necessitates the development and testing of new root canal medications with fewer side effects and more biocompatibility.

Herbal medications have been used as antibacterial, analgesic, and anti-inflammatory drugs with the benefits of being naturally renewable, having few adverse effects, being affordable, and being better tolerated by patients. The study of plant-based products for use as medicines is becoming more popular in the post-antibiotic era. Approximately one-third of newly approved small molecules derive from unaltered natural botanical products or their derivatives^9^. Medical folklore has used the Neem tree (*Azadirachta indica*) for centuries. People primarily cultivate it in the southern regions of Asia and Africa. Researchers have linked the leaves, fruit, blossoms, oil, gum, and other elements of the tree to positive health effects and have used them to cure a variety of illnesses, such as heart disease, diabetes, and hypertension. Moreover, these tree elements possesse antibacterial, anti-inflammatory, antioxidant, and anti-cancer properties^10^.

In recent times, research has demonstrated that *Azadirachta indica* is rich in numerous bioactive compounds, and many of them could offer pharmacological potential. There are more than three hundred distinctive compounds identified in the Neem tree. Some of the plentiful phytochemicals include gedunin, azadirachtin, nimbolide, and limonoids^11^. Researchers have previously isolated other compounds from neem seed oil and investigated their antioxidant and anti-inflammatory properties. These compounds include nimbolide, nimbin, nimbidin (triterpenoids), azadirachtin, and gedunin^12^. Recently, Beta-d-Mannofuranoside has also been identified as a promising antibacterial bioactive component of neem^13^. Encouraging data suggest the efficacy of neem in inhibiting bacterial growth and targeting bacterial biofilms, which makes it a promising source for drug discovery^11^.

The rise of nanotechnology has provided new antimicrobial alternatives. Nanoparticles (NPs) have earned lots of interest due to their bactericidal effects and are currently increasingly used to target bacterial infections as an antibiotic alternative^14^. Researchers are increasingly investigating the medicinal powers of nanoparticulate systems as potential antimicrobial agents. These systems exhibit a significant antimicrobial action, especially against bacterial infections^15^. They help in fighting microbes using multiple mechanisms simultaneously and could also act as good antimicrobial carrier^16^. Nanoparticles (NPs) are sub-micron suspensions with particles ranging in size from 1 to 100 nm with a high surface-to-volume ratio. This allows for stronger and more specific interactions with the bacterial cell wall at lower dosages, resulting in increased antibacterial activity. The narrow particle size distribution facilitates passage across bacterial cells^17^. Nanoparticles increase medication solubility and stability. They can penetrate membranes via endocytosis or through interactions with surface lipids. Nanoparticles show broad bactericidal activity and are used as carriers for the transport of antimicrobials. The antibacterial action of nanoparticles may be attributed to reactive oxygen species (ROS) generation that induce oxidative stress. It may also result from cell membrane disruption, interruption of electron transport chain, bacterial DNA damage, and damage to protein synthesis machinery^18^.

Nanoparticles labeled with antibacterials help in achieving co-localization of the antibacterial substance and also encourage the binding of antibacterial substances at the site of interaction^19^. Nano-antimicrobials have an inner antimicrobial activity represented in nanoparticles and additionally enhance the overall effectiveness of the enclosed antimicrobials by acting as nanocarriers. They help to achieve the desired beneficial effect by improving bioavailability at low doses. As a result, the drug’s dose-dependent side effects and toxic effects can be reduced. Additionally, the drug will be only released at the site of action in a sustained manner, thus delivering the maximum amount of drug to the infected site and achieving the best antimicrobial effect^20^. Using nanoparticles would additionally help in decreasing the therapeutic concentrations of drug compounds, including herbal medications. This helps to achieve a more potent effect, which in turn helps to decrease the toxic effect of herbal medicines.

There is a growing body of evidence about the potential of neem as a combinatorial or alternative antimicrobial agent in dentistry. Not much data is available about the possible use of nanoparticles in root canal infections, and to the best of our knowledge, no data is available about the use of Neem NPs in this field. In this work, we aim to investigate the potential of neem as an intracanal medication and to test the efficacy of its nano formulations. We also compare this effect to conventional Ca (OH)_2_ treatment, including its nano formulations. We also assess the biofilm forming capacity of *Mutans Streptococci* clinical isolates and the anti-biofilm effect of Neem oil and calcium hydroxide as compared to nanoparticle preparations of the same compounds against different tested clinical isolates.

## Results

### Identification of *Streptococcus* and *Enterococcus* clinical isolates

**Fig. 1:**
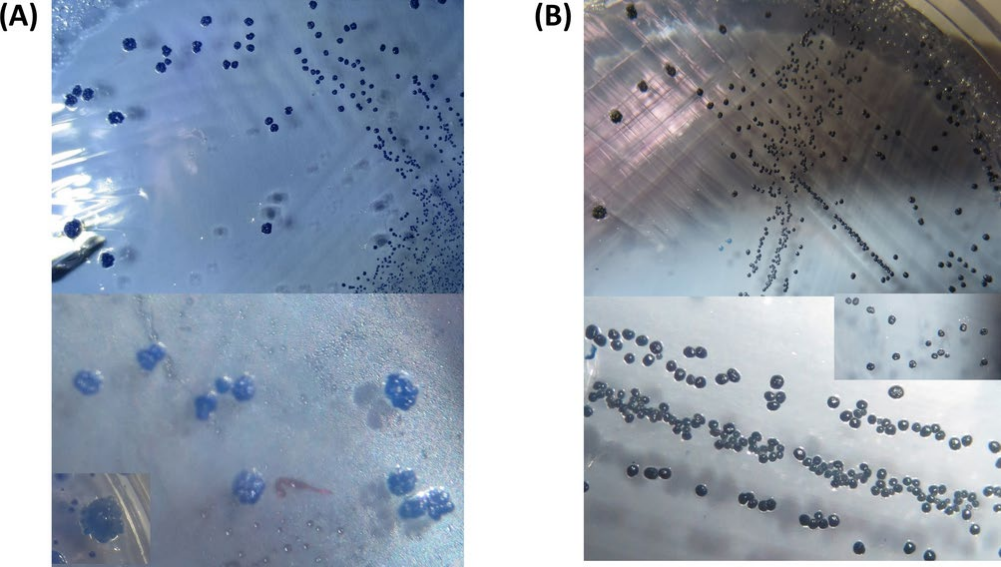
Colonial morphologies of *Mutans Streptococci* group and *E. faecalis* observed on Mitis salivarius bacitracin (MSB) agar. The most common morphotype was a typical granular light- blue to black blue "frosted-glass" appearance sometimes with a creamy marzipan consistency (**A**), *Enterococcus faecalis* showed small black colonies on MS agar (**B**).

**Fig. 2:**
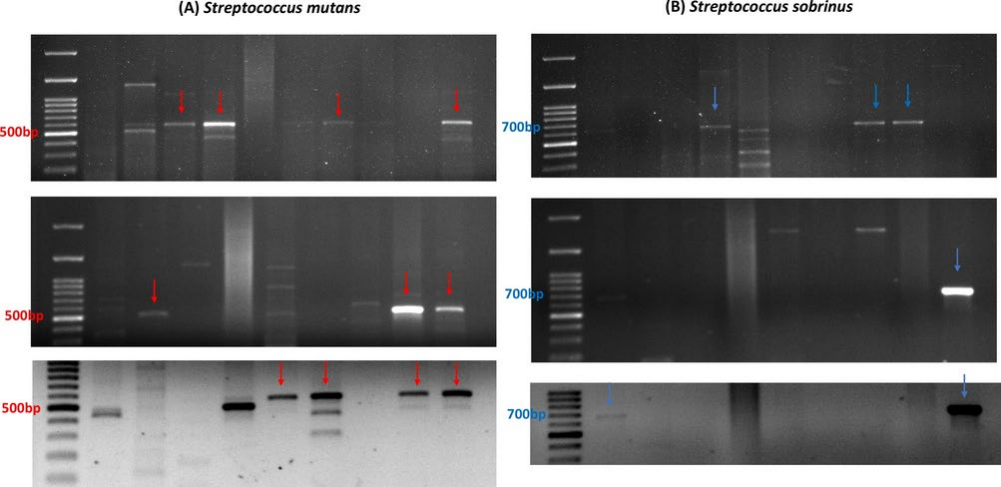
Identification of *S. mutans* and *S. sobrinus* using PCR. GTFB-F and GTFB-R were designed to amplify a 517-bp DNA fragment of the *gtf* B sequence of *S. mutans* (**A**) and primers GTFI-F and GTFI-R were designed to amplify a 712-bp DNA fragment of the *gtf* I sequence of *S. sobrinus* (**B**)*.* Full-length Plots are provided in Supplementary Material (Supplementary Figs. 5A,B).

After a three-day incubation period under a CO_2_-enriched atmosphere at 37 ℃, the agar plates were inspected for growth of *Mutans Streptococci* colonies. Pure cultures of each isolate were identified by their capacity to ferment different sugars, arginine hydrolyses, hydrogen peroxide and catalase production. A total of 34 isolates were identified from a total of 28 endodontic root canal samples. The isolated strains included 19 *S. mutans*, 13 *S. Sobrinus* and 2 *E. faecalis* strains. Isolated strains were identified based on the phenotypic morphology of growth on modified Mitis Salivarius (MS) agar and based on their biochemical reactions. Colonies of *S. mutans* showed typical granular light blue to dark blue "frosted-glass" appearance while *E. faecalis* colonies showed small black colonies on MS agar (Fig. 1). *E. faecalis* were identified as Gram-positive cocci, non-motile, catalase negative, oxidase negative showing positive nitrate reduction, positive arginine hydrolysis, positive bile esculin hydrolysis and positive growth in 6.5% NaCl broth. PCR was then used to confirm the identity of isolated *Mutans Streptococci* species. Figure 2 shows PCR identification of *S. mutans* and *S. sobrinus* isolates.

### Characterization of nano formulations

Measurement of particle size and zeta potential determination showed 163.3 ± 9.77 nm particle size, 0.322 ± 0.028 polydispersity index (PDI), and −12.2 ± 1.3 mV Zeta potential for Ca (OH)_2_ NPs. For Neem oil NPs, it showed 178.1 ± 10.02 nm particle size, 0.36 ± 0.030 PDI, and − 32.4 ± 0.77 mV Zeta potential.

Graphs showing Size Distribution Report by Intensity and Zeta potential for the three replicates tested for each preparation are provided in Supplementary Figs. 1, 2.

### Antimicrobial efficacy of neem oil and neem NPs preparations against isolated strains

**Fig. 3:**
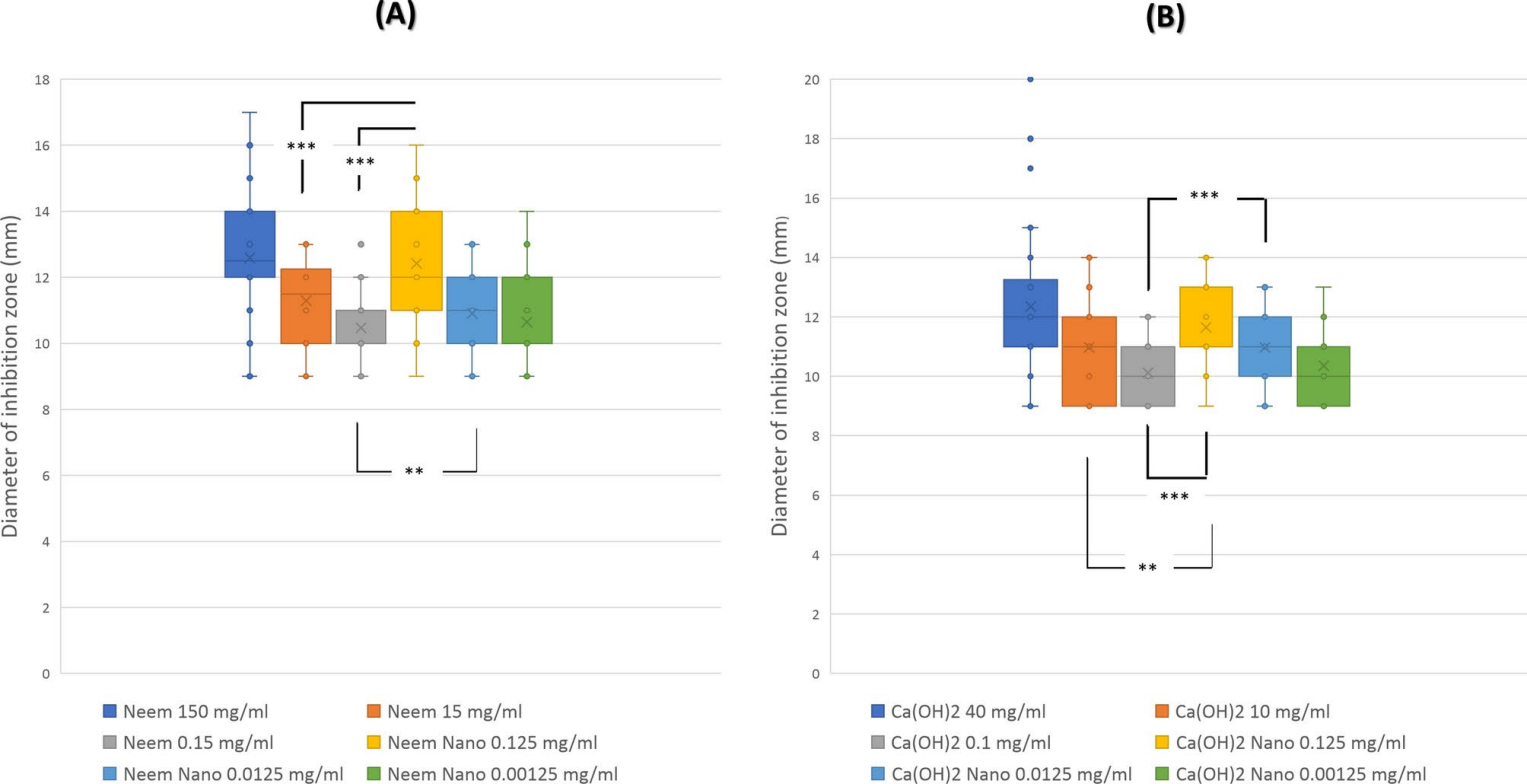
The distribution of the diameters of inhibition zones for each of the tested. Neem oil, Neem oil NPs, Ca (OH)_2_ , and Ca (OH)_2_ NPs. Neem 150 mg/ml: Mean (SD) 12.58 (1.77), Neem Nano 0.125 mg/ml: Mean (SD) 12.4 (1.67), Neem 15 mg/ml: Median (IQR) 11.5 (10–12) , Neem 0.15 mg/ml: Median (IQR) 11 (10–11) , Neem Nano 0.0125 mg/ml: Median (IQR) 11 (10–12) , and Neem Nano 0.00125 mg/ml: Median (IQR) 10 (10–11.75), Nano Ca(OH)_2_ 0.0125 mg/ml: Mean (SD) 10.97 (1.167), Ca(OH)_2_ 40 mg/ml: Median (IQR) 12 (11–13) , Ca(OH)_2_ 10 mg/ml: Median (IQR) 11 (9.25–12) , Ca(OH)_2_ 0.1 mg/ml: Median (IQR) 10 (9–11), Nano Ca(OH)_2_ 0.125 mg/ml: Median (IQR) 11 (11–13) , Nano Ca(OH)_2_ 0.00125 mg/ml: Median (IQR) 10 (9–11). *P* values: ** < 0.05, *** < 0.0001.

Shapiro- Wilk test showed that both Neem 150 mg/ml and Neem NPs 0.125 mg/ml were normally distributed (*p* = 0.224 and *p* = 0.152 respectively). All other tested concentrations including Neem 15 mg/ml, Neem 0.15 mg/ml, Neem NPs 0.0125 mg/ml, and Neem NPs 0.00125 mg/ml did not show normal distribution. Figure 3A shows the distribution of the diameters of inhibition zones for each of the tested Neem oil and Neem oil NPs preparations.

The results of Friedman test indicated that there was a statistically significant difference in the diameter of inhibition zones across the six Neem and Neem NPs concentrations tested χ^2^ (5, n = 34) = 80.166, *p* < 0.0001). Inspection of median values showed the highest zone diameter mean rank from the Neem 150 mg/ml (*Md* = 12.5), this was followed by zones from Neem NPs 0.125 mg/ml (*Md* = 12) which was better than both Neem 15 mg/ml (*Md* = 11.5) and Neem 0.15 mg/ml (*Md* = 11). Notably, also that each of Neem NPs 0.0125 mg/ml (*Md* = 11) and Neem NPs 0.00125 mg/ml (*Md* = 10) showed larger zone diameter and higher mean rank than Neem 0.15 mg/ml (*Md* = 11). Post-hoc testing using Wilcoxon signed rank test using Bonferroni adjusted alpha values for test comparisons ( α = 0.0125) showed that the difference in zone diameter between Neem NPs 0.125 mg/ml and each of Neem 15 mg/ml and Neem 0.15 mg/ml were statistically significant ( *p* < 0.0001 for both) (*z* = −3.593 and *z* =  −4.411 respectively) with medium and large effect size respectively (*r* = 0.44, *r* = 0.54).

The difference between Neem NPs 0.0125 mg/ml and each of Neem 15 mg/ml and Neem 0.15 mg/ml were statistically insignificant (*p* = 0.072 and *p* = 0.037 respectively), (*z* = −.801 and *z* = −2.081 respectively) with small effect size (*r* = 0.22 and *r* = 0.25 respectively). Supplementary Fig. 3A shows Disc Diffusion antimicrobial susceptibility inhibition zones for different neem and neem NPs tested preparations.

### Antimicrobial efficacy of Ca (OH)_2_ and Ca (OH)_2_ NPs preparations against isolated strains

Shapiro- Wilk test showed that most tested concentrations of Ca (OH)_2_ and Ca (OH)_2_ NPs preparations were non-normally distributed. Figure 3 shows the distribution of the diameters of inhibition zones for each of the tested Ca (OH)_2_ and Ca (OH)_2_ NPs preparations.

The results of Friedman test indicated that there was a statistically significant difference in the diameter of inhibition zones across the six Ca (OH)_2_ and Nano Ca (OH)_2_ concentrations tested χ^2^ (5, n = 34) = 75.105, *p* < 0.0001). Inspection of median values showed the highest zone diameter mean rank from the Ca (OH)_2_ 40 mg/ml (*Md* = 12), this was followed by zones from Ca (OH)_2_ NPs 0.125 mg/ml (*Md* = 11) which was better than Ca (OH)_2_ 10 mg/ml (*Md* = 11). Also, Ca (OH)_2_ NPs 0.0125 mg/ml (*Md* = 11) showed larger zone diameter and higher mean rank than each of Ca(OH)_2_ 10 mg/ml (*Md* = 11) and Ca(OH)_2_ 0.1 mg/ml (*Md* = 10) . Post-hoc testing using Wilcoxon signed rank test using Bonferroni adjusted alpha values for four test comparisons ( α = 0.0125) showed that that difference in zone diameter between Ca(OH)_2_ NPs 0.125 mg/ml and each of Ca(OH)_2_ 10 mg/ml and Ca(OH)_2_ 0.1 mg/ml were statistically significant ( *p* = 0.008, *p* < 0.0001 respectively) (*z* =  − 2.67 and *z* =  − 4.602 respectively) with medium and large effect size respectively (*r* = 0.32, *r* = 0.56). The difference between Ca (OH)_2_ NPs 0.0125 mg/ml and Ca (OH)_2_ 10 mg/ml was statistically insignificant (*p* = 0.847, *z* =  − 0.193 with weak effect size (*r* = 0.02) while that between Ca (OH)_2_ NPs 0.0125 mg/ml and Ca (OH)_2_ 0.1 mg/ml was statistically significant (*p* < 0.0001, *z* =  − 3.863 with medium effect size (*r* = 0.47). Supplementary Fig. 3B shows Disc Diffusion antimicrobial susceptibility inhibition zones for different Ca (OH)_2_ and Ca (OH)_2_ NPs tested preparations.

### Antibiofilm effect of neem oil and neem oil NPs preparations against isolated strains

**Fig. 4:**
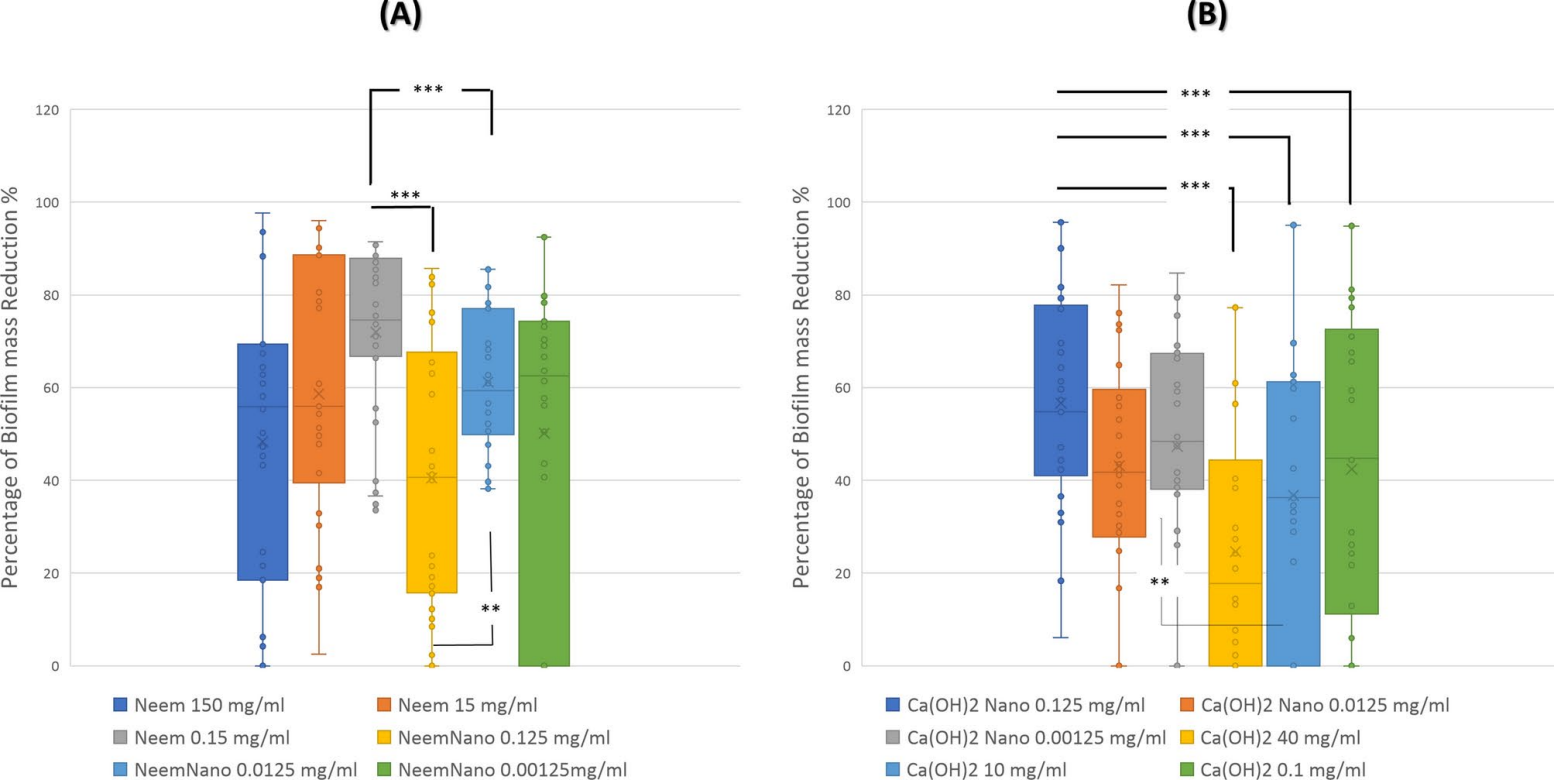
The percentage of biofilm mass reduction for each of the tested. Neem oil, Neem oil NPs, Ca (OH)_2_ , and Ca (OH)_2_ NPs. Neem Nano 0.0125 mg/ml: Mean (SD) 61.23 (15.3), Neem 150 mg/ml: Median (IQR) 55.9 (19.3–68.9), Neem 15 mg/ml: Median (IQR) 55.9 (41.5–86.5) , Neem 0.15 mg/ml: Median (IQR) 74.5 (67.4–87.6) , Neem Nano 0.125 mg/ml: Median (IQR) 40.6 (16.1–64.8) , and Neem Nano 0.00125 mg/ml: Median (IQR) 62.5 (10.2–74), Ca (OH)_2_ 40 mg/ml: Mean (SD) 25 (25.1) and Ca (OH)_2_ 10 mg/ml: Mean (SD) 36.8 (29), Ca (OH)_2_ 0.1 mg/ml: Median (IQR) 44.7 (13.1–70.1), Nano Ca (OH)_2_ 0.125 mg/ml: Median (IQR) 54.7 (42.4–77.2) , Nano Ca(OH)_2_ 0.0125 mg/ml: Median (IQR) 41.7 (28.7–57.8), Nano Ca(OH)_2_ 0.00125 mg/ml: Median (IQR) 48.4 (38.8–67.1). *P* values: ** < 0.05, *** < 0.0001.

Shapiro- Wilk test showed that antibiofilm results for all tested concentrations of Neem and Neem NPs preparations were non-normally distributed except for Neem NPs 0.0125 mg/ml (*p* = 0.058). Figure 4A shows the percentage of biofilm mass reduction for each of the tested Neem oil and Neem oil NPs preparations.

The results of Friedman test indicated that there was a statistically significant difference in the percentage of biofilm mass inhibition across the six Neem and Neem NPs concentrations tested χ^2^ (5, n = 34) = 32.681, *p* < 0.0001). Inspection of median values showed the highest biofilm mass inhibition mean rank from the Neem 0.15 mg/ml (*Md* = 74.55), this was followed by high biofilm mass inhibition by Neem NPs 0.0125 mg/ml (*Md* = 59.33). This was followed by Neem 15 mg/ml (*Md* = 56) and Neem NPs 0.00125 mg/ml (*Md* = 62.5) which showed higher mean rank than Neem 150 mg/ml (*Md* = 55.9) and Neem NPs 0.125 mg/ml (*Md* = 40.6).

Post-hoc testing using Wilcoxon signed rank test using Bonferroni adjusted alpha values for test comparisons ( α = 0.0125) showed that that difference in biofilm mass reduction between Neem 0.15 mg/ml and each of Neem NPs 0.125 mg/ml, Neem NPs 0.0125 mg/ml, and Neem NPs 0.00125 mg/ml were statistically significant ( *p* < 0.0001 for the three pair comparisons), (*z* = -−4.66, *z* = -−4.044, *z* = -−4.471, respectively) with large effect size (*r* = 0.56, *r* = 0.49, *r* = 0.54, respectively). Additionally, the difference between Neem NPs 0.125 mg/ml and Neem NPs 0.0125 mg/ml was statistically significant, *z* = -−3.155, *p* = 0.002 with medium effect size (*r* = 0.38).

### Antibiofilm effect of Ca (OH)_2_ and Ca (OH)_2_ NPs preparations against isolated strains

Shapiro- Wilk test showed that antibiofilm results for all tested concentrations of Ca (OH)_2_ and Ca (OH)_2_ NPs preparations were non-normally distributed except for Ca (OH)_2_ 40 mg/ml and Ca (OH)_2_ 10 mg/ml which were normally distributed (*p* = 0.471 and *p* = 0.135 respectively). Figure 4B shows the percentage of biofilm mass reduction for each of the tested Ca (OH)_2_ and Ca (OH)_2_ NPs preparations.

The results of Friedman test indicated that there was a statistically significant difference in biofilm mass reduction across the six Ca (OH)_2_ and Ca (OH)_2_ NPs concentrations tested χ^2^ (5, n = 34) = 61.509, *p* < 0.0001). Inspection of median values showed the highest biofilm mass inhibition mean rank from the Ca (OH)_2_ NPs 0.125 mg/ml (*Md* = 54.7) which was followed by Ca (OH)_2_ NPs 0.00125 mg/ml (*Md* = 48.4). Ca (OH)_2_ 0.1 mg/ml (*Md* = 44.7) showed higher biofilm mass inhibition and higher mean rank than each of Ca (OH)_2_ 10 mg/ml (*Md* = 36.3) and Ca (OH)_2_ 40 mg/ml (*Md* = 17.7) which showed the lowest effect on biofilm mass inhibition.

Post-hoc testing using Wilcoxon signed rank test using Bonferroni adjusted alpha values for test comparisons ( α = 0.0125) showed that that difference biofilm mass reduction between Ca(OH)_2_ NPs 0.125 mg/ml and each of Ca(OH)_2_ 40 mg/ml, Ca(OH)_2_ 10 mg/ml, and Ca(OH)_2_ 0.1 mg/ml were statistically significant (*p* < 0.0001 for the three pair wise comparisons), (*z* = -−5.087, *z* = -−5.087, *z* = -−3.6, respectively) with large and medium effect sizes (*r* = 0.62, *r* = 0.62, *r* = 0.44, respectively). The difference in antibiofilm effect between Ca (OH)_2_ NPs 0.00125 mg/ml and Ca (OH)_2_ 10 mg/ml was also statistically significant, *z* = -2.685, *p* = 0.007 with medium effect size (*r* = 0.33).

**Fig. 5:**
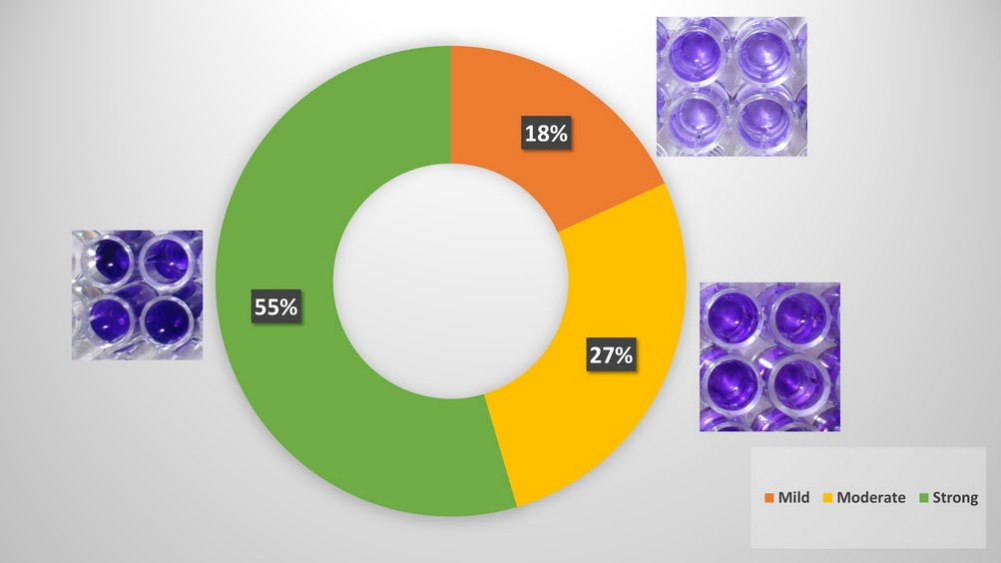
Distribution of tested isolates according to their biofilm formation capacity.

Figure 5 shows classification of tested isolates according to their biofilm formation capacities.

### Interaction of neem components with *Streptococcus mutants* surface proteins

From the in-silico docking results, Sitosterol showed the best binding affinity to *Streptococcus mutants* proteins among compounds tested. It showed the highest binding affinity to *Streptococcus mutans* GtfB, Glucosyltransferase-I (−9.86), *Streptococcus mutans* antigen I/II (AgI/II) protein adhesin (−9.85), Peptidase Domain of *Streptococcus mutans* ComA (−9.74), and Glucan Binding Protein C of *Streptococcus mutans* (−9.69). Additionally, *Streptococcus mutans* antigen I/II (AgI/II) protein adhesin was the best binding protein to each of Gedunin and Nimbin with binding affinities of (−9.46) and (−7.67) respectively. Quercetin showed highest binding affinity to Glucan Binding Protein C of *Streptococcus mutans* (−6.29). Beta-d-Mannofuranoside, O-geranyl showed the lowest binding affinity to *Streptococcus mutans* proteins among compounds tested and its highest binding affinity was achieved with *Streptococcus mutans* GtfB, Glucosyltransferase-I (−4.5). Tables 1, 2 list the results of all tested macromolecules with neem active compounds. Supplementary Fig. 4 shows the best docking poses and interaction bonds for the highest binding affinity observed for each of the tested neem active components.

**Table 1:** Interaction and binding affinity of Nimbin, Gedunin, and Quercetin with *S. mutans* surface proteins.

Ligand (Neem active component)	Macromolecule ( *S. mutans* protein)	Reference RMSD	Binding energy (Kcal/mol)	Inhibition constant (Ki)	No of H bonds (drug-protein)	Amino acid involved in interaction
Nimbin	*S. mutans* antigen I/II major cell-surface adhesin (3QE5)	175.170 A	−4.84	282.31 uM	2	ILE 1227B, GLU 1234B
	*S. mutans* antigen I/II cell surface-localized protein adhesin (3IOX)	38.014 A	−7.67	2.40 uM	3	GLU 701A, SER 704A, LYS 811A
	*atm*B (putative membrane lipoprotein) (4Q5T)	51.492 A	−6.96	7.97 uM	3	TYR 89A, ALA 107A, GLU 108A
	*S. mutans* adhesin P1 (4TSH)	58.227 A	−4.64	395.81 uM	2	ALA 70A, THR 1118B
	Glucan binding protein C (5UQZ)	78.956 A	−6.86	9.29 uM	2	ASN 442A, THR 449A
	Peptidase domain of ComA (5XE8)	36.527 A	−6.58	15.08 uM	2	MET 72C, GLN 128A
	Glucan binding protein C (6CAM)	229.709 A	−6.45	18.68 uM	3	ILE 219A, LEU 220A (2)
	SspB Agglutinin receptor (6Q2L)	18.169 A	−5.42	106.22 uM	2	ASN 717A, GLY 755A
	Collagen binding protein CNM (7LGR)	32.638 A	−5.9	47.74 uM	3	SER 313A (2), ALA 315A
	Streptococcus mutans GtfB (8FKL)	51.143 A	−6.48	17.85 uM	2	ASP 450A, GLY 451A
Gedunin	*S. mutans* Antigen I/II major cell-surface adhesin (3QE5)	188.481 A	−6.99	7.48 uM	2	ALA 1034A, ASP 1035A
	*S. mutans* antigen I/II cell surface-localized protein adhesin (3IOX)	35.962 A	−9.46	116.67 nM	2	SER 704A, TRP 816A
	*atm*B (putative membrane lipoprotein) (4Q5T)	47.277 A	−7.87	1.69 uM	1	TYR 89A
	*S. mutans* adhesin P1 (4TSH)	48.372 A	−5.94	44.55 uM	1	ILE 1326B
	Glucan binding protein C (5UQZ)	76.612 A	−8.64	465.25 nM	2	TRP351A, THR449A
	Peptidase domain of ComA (5XE8)	40.382 A	−8.03	1.30 uM	3	LYS 69C, ARG 126A (2)
	Glucan binding protein C (6CAM)	47.292 A	−7.9	1.61 uM	1	TYR 89A
	SspB agglutinin receptor (6Q2L)	17.510 A	−7.75	2.08 uM	2	SER 648A, TRP 744A
	Collagen binding protein CNM (7LGR)	27.787 A	−7.73	2.14 uM	1	THR 226A
	*Streptococcus mutans* GtfB (8FKL)	50.426 A	−8.69	423.49 nM	3	ASP 450A, GLY 451A, PHE 650A
Quercetin	*S. mutans* Antigen I/II Major cell-surface adhesin (3QE5)	39.512 A	−5.71	64.72 uM	8	ASN 516A, GLN 592A, LYS 594A, THR 649A (3), ASP 650A
	*S. mutans* antigen I/II cell surface-localized protein adhesin (3IOX)	39.245 A	−5.81	54.76 uM	9	ASN 516A (2), GLN 592A, LYS 594A, THR 649A (3), ASP 650A
	*atm*B (putative membrane lipoprotein) (4Q5T)	50.830 A	−5.98	41.10 uM	8	THR38A (3), TRP47A, ASP68A (2), SER85A, TYR110A
	*S. mutans* adhesin P1 (4TSH)	90.310 A	−3.38	3.33 mM	4	GLU 132A, LYS 136A, ASP 1041B (2)
	Glucan binding protein C (5UQZ)	69.098 A	−6.29	24.42 uM	6	THR 300A, SER 346A (2), ASP 413A, ALA 453A (2)
	Peptidase domain of ComA (5XE8)	38.022 A	−6.04	37.16 uM	6	LYS 69A, LYS 69C, ALA 70A (2), ASP 71C, GLN 128A
	Glucan binding protein C (6CAM)	50.545 A	−5.99	40.47 uM	7	THR 38A (3), TRP 47A, ASP 68A (2), TYR 209A
	SspB agglutinin receptor (6Q2L)	15.279 A	−5.66	71.01 uM	10	ASN 645A, ASP 710A (4), ASN 717A, ASN 748A, ASN 750A, ASN 752A (2)
	Collagen binding protein CNM (7LGR)	30.644 A	−4.89	260.68 uM	7	THR 178A, ASP 180A, MET 181A, HIS 312A, SER 313A, ALA 315A (2)
	*Streptococcus mutans* GtfB (8FKL)	50.419 A	−5.45	101.09 uM	6	PRO 681A, TYR 683A, SER 690A, LYS 712A, PRO 713A (2)

**Table 2:** Interaction and binding affinity of Sitosterol and beta-d-Mannofuranoside, O-geranyl with *S. mutans* surface proteins.

Ligand (Neem active component)	Macromolecule (*S. mutans* protein)	Reference RMSD	Binding energy (Kcal/mol)	Inhibition constant (Ki)	No of H bonds (drug-protein)	Amino acid involved in interaction
Sitosterol	*S. mutans* Antigen I/II Major cell-surface adhesin (3QE5)	189.605 A	−5.89	48.25 uM	2	GLU 1007A, LYS 1032A
	*S. mutans* antigen I/II cell surface-localized protein adhesin (3IOX)	37.073 A	−9.85	60.25 nM	14	LEU 553A, ASP 554A, THR 586A, VAL 587A, PHE 656A (2), LYS 811A, LYS 812A (3), ILE 815A (2), TRP 816A (2)
	*atm*B (putative membrane lipoprotein) (4Q5T)	48.373 A	−8.9	298.79 nM	9	VAL 106A, TRP 243A (2), LYS 253A, ILE 256A (3), LYS 275A, HIS 260A
	S. mutans adhesin P1 (4TSH)	190.639 A	−6.76	11.07 uM	1	THR 1470B
	Glucan binding protein C (5UQZ)	71.046 A	−9.69	78.81 nM	2	THR 449A (2)
	Peptidase domain of ComA (5XE8)	61.112 A	−9.74	72.33 nM	8	LEU 22C, ALA 58C, PHE 63C (3), LEU 138C (2), PRO 140C
	Glucan binding protein C (6CAM)	227.777 A	−8.11	1.13 uM	9	LEU 220A (2), TRP 351A (2), VAL 410A (2), LEU 446A, ILE 450A, TRP 451A
	SspB agglutinin receptor (6Q2L)	16.139 A	−8.46	632.67 nM	1	ASN 742A
	Collagen binding protein CNM (7LGR)	21.328 A	−6.74	11.48 uM	1	ASN 311A
	*Streptococcus mutans* GtfB (8FKL)	50.880 A	−9.86	59.23 nM	1	GLU 406A
beta-d-Mannofuranoside, O-geranyl	*S. mutans* Antigen I/II Major cell-surface adhesin (3QE5)	147.727 A	−1.53	75.57 mM	4	ILE 1157A (4)
	*S. mutans* antigen I/II cell surface-localized protein adhesin (3IOX)	38.455 A	−3.24	4.22 mM	6	GLU 701A (2), SER 704A, LYS 811A (2), LYS 812A
	*atm*B (putative membrane lipoprotein) (4Q5T)	62.023 A	−3.38	3.31 mM	5	ASP 41A (4), LYS 44A
	S. mutans adhesin P1 (4TSH)	90.269 A	−1.28	114.75 mM	4	ASP 1041B (2), ASP 1041B, GLU 1042B
	Glucan binding protein C (5UQZ)	109.890 A	−3.91	1.35 mM	2	GLU 472A (2)
	Peptidase domain of ComA (5XE8)	59.506 A	−4.16	887.25 uM	3	LEU 22C (3)
	Glucan binding protein C (6CAM)	231.135 A	−2.31	20.36 mM	4	ASN 349A, ASP 408A, THR 449A (2)
	SspB agglutinin receptor (6Q2L)	14.897 A	−3.86	1.49 mM	2	ASN 717A
	Collagen binding protein CNM (7LGR)	34.017 A	−3.4	3.24 mM	4	SER 313A (3), ALA 315A
	*Streptococcus mutans* GtfB (8FKL)	54.608 A	−4.5	500.75 uM	4	TYR 657A (4)

## Discussion

Neem products have been studied in dentistry for their potential to prevent or treat oral disease. Neem extracts have been studied for their ability to promote oral hygiene and to act as irrigators for root canals. Previous research reports have shown that *A. indica* has antibacterial efficacy against some common endodontic infections^21^. Also *A. indica* methanolic extract has previously demonstrated strong in vitro antibacterial activity against polymicrobial dental biofilm^22^. It has also been demonstrated that toothpastes or gels containing neem can lower oral *S. mutans* levels and possibly lessen gingivitis and plaque^23,22^. On the other hand, some other studies may report that other plants may show a superior effect against certain bacterial species including *S. mutans* and *E. faecalis*^24,25^.

Earlier studies have reported that Neem leaves aqueous extract could achieve an antibacterial effect against *S. mutans* in a concentration dependent manner^26^. Other studies have reported that ethanolic extract of neem leaves at 10 mg/ml used for antimicrobial assay could achieve a statistically significant better antimicrobial effect against *S. mutans* than Chlorhexidine^27^. However, other previous studies have reported that 2% chlorhexidine showed a statistically significant reduction in microorganisms count greater than 75 mg/ml of the methanolic plant extracts^28^. Another study has shown that neem aqueous extract achieved a higher antibacterial effect than both chlorhexidine and cloves against *S. mutans* (ATCC 25175) with a measured MIC of 4.2 mg/ml^29^. On the other hand, some other studies have demonstrated a superior antimicrobial effect achieved by Ca (OH)_2_ when compared to 5% of neem aqueous extract in a gel form against *E. faecalis*^30^. However, it is important to consider that there is some evidence showing that neem oil may show moderate cytotoxicity to fibroblasts at 0.5% to 0.25 mg/ml concentration^31^. Additionally, Ca (OH)_2_ showed moderate fibroblast cytotoxicity at concentration of 0.5 mg/mL which was reduced at 0.25 mg/ml and 0.125 mg/ ml Ca (OH)_2_ concentrations showing slight cytotoxicity^31^. These variable results have motivated us to examine the effect of both Neem NPs preparations and Ca (OH)_2_ NPs which would allow using lower effective concentration of the medication used with the aim of achieving better therapeutic effect and lower cytotoxicity.

In the current study, we aimed to explore the antibacterial and antibiofilm potential of neem oil and its nanoparticles preparations against some of the most common root canal endodontic pathogens isolated including *Mutans Streptococci* and *E. faecalis*. Results of the study showed that Neem NPs 0.125 mg/ml has a better antibacterial effect than each of Neem 15 mg/ml and Neem 0.15 mg/ml and the differences were statistically significant. In addition, each of Neem NPs 0.0125 mg/ml and Neem NPs 0.00125 mg/ml showed larger zone diameter and higher mean rank than Neem 0.15 mg/ml. This would enable using the compound at lower concentrations which result in reducing its cytotoxicity.

Regarding biofilm mass inhibition, highest biofilm mass inhibition mean rank was observed with Neem 0.15 mg/ml which was followed by Neem NPs 0.0125 mg/ml. Unexpectedly, higher concentration of Neem NPs 0.125 mg/ml showed the lowest mean rank biofilm mass inhibition while Neem NPs 0.0125 mg/ml showed a better inhibitory effect, and the difference was statistically significant. The three tested neem NPs concentrations showed variable antibiofilm effects which indicates that the viscosity and particle size of nanopreparations could affect its diffusion into biofilm material. The lower antibiofilm effect of higher concentrations of neem NPs (0.125 mg/ml) may be attributed to higher viscosity of the tested preparations which may technically interfere with measurement of antibiofilm effect as a result of adherence to polyestrene wells which can be falsely interpreted as biofilm formation as a result of adherence and subsequent crystal violet staining of tested preparations. Considering such a limitation, future testing of antibiofilm effect using other methods including confocal laser scanning microscopy for mature biofilm inhibition is recommended.

Ca (OH)_2_ NPs may offer a better alternative to conventional Ca (OH)_2_ medication. It may be expected that reducing the size of Ca (OH)_2_ particles into nanoparticles may enhance the penetration of this medicament into dentinal tubules and increase their antimicrobial efficacy, however, to the best of our knowledge, this has not been well-studied. Recent studies have demonstrated improvement of sealability of experimental nano calcium hydroxide sealer with significantly less dye leakage and have also shown the ability of calcium hydroxide nanoparticles to enhance the physical properties of root canal sealer, however, their antibacterial and antibiofilm effect has not been studied^32,33^.

In the current study, Nano Ca (OH)_2_ 0.125 mg/ml showed statistically significant better antibacterial effect than Ca (OH)_2_ 10 mg/ml and Ca (OH)_2_ 0.1 mg/ml. Additionally, Nano Ca (OH)_2_ 0.0125 mg/ml showed larger zone diameter and higher mean rank than each of Ca (OH)_2_ 10 mg/ml and Ca (OH)_2_ 0.1 mg/ml. In addition, Ca (OH)_2_ NPs showed a pronounced antibiofilm effect. Nano Ca (OH)_2_ 0.125 mg/ml showed a statistically significant higher antibiofilm effect than each of Ca (OH)_2_ 40 mg/ml, Ca (OH)_2_ 10 mg/ml, and Ca (OH)_2_ 0.1 mg/ml. Also, Ca (OH)_2_ 0.1 mg/ml showed higher biofilm mass inhibition and higher mean rank than each of Ca (OH)_2_ 10 mg/ml and Ca (OH)_2_ 40 mg/ml which showed the lowest effect on biofilm mass inhibition. Different factors can affect uptake of nanoparticles into the biofilm material and into the cell wall of the bacteria including type and size of particles in addition to PH change. Better antibiofilm effect was observed in Ca (OH)_2_ nanoparticles as compared to neem nanoparticles at same concentration which may indicate that PH change could affect the uptake and diffusability of Ca (OH)_2_ nanoparticles into biofilm material offering an advantage to Ca (OH)_2_ NPs.

Finding important active substances and components of neem and identifying important binding sites can aid in the process of structure-based drug discovery through suggesting changes that can improve ligand affinity and avoiding changes that may clash with the protein receptor. Docking was performed to find out and screen for interactions between possible ligands in neem and potential protein receptors in *S. mutans.*

*Streptococcus mutans* antigen I/II (AgI/II) cell surface-localized protein adhesin was one of the best binding protein to multiple active compounds of Neem including Sitosterol, Gedunin and Nimbin with binding affinities of (−9.85), (−9.46) and (−7.67) respectively. Ag I/II has been investigated as an immunological target of protective immunity and is known to contribute to pathogenicity. It facilitates bacterial invasion of dentin, influences the formation of biofilms, and mediates attachment to the tooth surface^34^. It interacts with the salivary agglutinin glycoprotein complex on the surface of *S. mutans*, where it is located^35^.

Glucan Binding Protein C (GbpC) also showed high binding affinities to tested compound including Sitosterol with binding affinity of −9.69, Gedunin with binding affinity of −8.64, and Nimbin with binding affinity of −6.86. Unlike Ag I/II, it is considered an adhesin of *S. mutans* and a virulence factor dependent on sucrose. It shares structural resemblance with the V regions of AgI/II and a similar functional adherence to salivary agglutinin. However, GbpC was found to be distinctive in its tendency to adhere to dextran and glucans^36^.

A possible limitation that may affect interpretation of the study results is the practicality of measuring higher concentrations of Nano Neem preparations due to its high viscosity which was observed at concentration of 0.125 mg/ml Neem NPs preparations. The high viscosity resulted in the adherence of material to polyesterene which may have interfered with measurement of true biofilm adherence. Similarly, higher concentrations of Ca (OH)_2_ tend to precipitate, and this was observed at 40 mg/ml and 10 mg/ml Ca (OH)_2_concentrations. The precipitate could be adherent to polyesterene wells and stained resulting in false interpretation of higher degree of biofilm formation.

In this study, we highlight the promising antibacterial and antibiofilm potential of Neem oil including its nanoparticle formulation as an intracanal medicament. Moreover, we show that Ca (OH)_2_ nano preparations exhibit a superior antibacterial and antibiofilm effect compared to the conventional preparations and may offer a better alternative to traditional Ca (OH)_2_ used in endodontic treatments. Future studies may additionally test for these promising properties using additional approaches including viability stains, viability qPCR, confocal scanning laser microscopy, and fluorescence in situ hybridization. We also reveal the active neem constituents that exhibit the best binding affinities to different *S. mutans* surface proteins. In silico analysis showed that both Sitosterol and Gedunin appear to be important active constituents of neem and possible drug candidates. *Streptococcus mutans* antigen I/II (AgI/II) protein adhesin and Glucan Binding Protein C appear to be important drug targets. The information provided in this research may offer a guide for further development of neem as a medication for use in the treatment of root canal infections.

## Materials and methods

### Ethical approval

The study was approved by the Institutional Review Board and ethics committee of the Faculty of Dentistry, Suez Canal University, ethical committee approval’s No. 2024/787. An informed consent was obtained from participants to collect samples. Experiments were performed according to relevant guidelines and regulations.

### Sample collection

Single rooted Teeth with necrotic pulp and periapical lesion were selected for root canal sampling. Teeth were isolated with rubber dam and disinfected with 30% H_2_O_2_, and 2.5%NaOCl to assure aseptic conditions before any procedure, Sodium thiosulphate was then used to deactivate NaOCl. The pulp chamber was accessed, then size 10 K-file (Mani, Inc., Tochigi, Japan) was inserted into the root canal. Sterile water was deposited with hypodermic syringe, and a 27-gauge needle and the contents were debrided from canal walls by push–pull motion. Then, root canal sample was taken with presterilized paper points. Two paper points were placed in the canal for 60 s and then transferred into presterilized tubes of 2 ml brain heart infusion broth (BHI broth). These tubes were then transferred within 4 h to Microbiology laboratory for culturing under aerobic and anaerobic conditions. Thirty-Five samples were collected. A total of 34 isolates were identified from a total of 28 endodontic root canal samples showing positive growth. The identified strains included 19 *S. mutans*, 13 *S. Sobrinus* and 2 *E. faecalis* strains and all were further tested.

### Microbiologic processing for isolation of Mutans-*Streptococcus* and *Enterococcus* species

Collected samples were centrifuged and sediment cultured on MS agar and Modified MS bacitracin agar and incubated at 37 ℃ in both aerobic environment and in 5–10% CO^2^ for up to 72 h in a candle jar. Streptococcal species causing root canal infections were isolated from root canal samples using Mitis Salivarius (MS) agar and Modified MS bacitracin agar composed of Mitis-salivarius agar (MSA) supplemented with 200 U Bacitracin/L, 20% sucrose and 1% potassium tellurite in addition to 2 mg of gramicidin, 10 mg of colistin, and 10 mg of nalidixic acid per Litre and were further identified using colonial morphology and biochemical reactions. Cultured microorganisms were visualized after staining by Gram’s method. Further identification and confirmation of *S. mutans* and *S. sobrinus* were performed by PCR using species-specific primers.

In order to identify *Mutans Streptococci* and *Enterococcus* isolates, biochemical tests were performed according to the method described by Shklair and Keene^37^. A phenol red broth base was used as a basal medium for fermentation of different sugars tested. The sugars were sterilized using Millipore filtration (0.22 µm pore size) and were added aseptically to warm basal medium to a final concentration of 1%. The media were dispensed into sterile screw capped test tubes and inoculated with the tested organism and tubes were read after 48 h of incubation. Level of ammonia production from L-arginine was determined using the method described by Niven et al.^38^. After 48 h incubation, 0.1 ml Nassler’s reagent was directly added to the medium and production of ammonia was indicated by development of yellow–red color. The nitrate reduction test was performed by inoculating nitrate broth with 2 drops from a young broth culture of the test organism and incubated for 48 h at 35 ºC. Cultures are then tested 24 h after obvious growth is detected. Five drops of each of nitrate reagent solution A (sulfanilic acid) and B (alpha-naphthylamine) are added and then observed for at least 3 min for red color to develop indicating positive nitrate reduction^39^.

### Chromosomal DNA extraction

Cultures frozen at –70 ℃ were streaked on MSB agar and incubated for 48 h in in 5–10% CO^2^ in a candle jar at 37 ℃. Isolated pure colonies of the same morphology from each strain were transferred to brain–heart infusion broth and incubated overnight at 37 ℃ to stationary phase. The cells were harvested by centrifugation at 4,000 rpm for 15 min and washed twice with phosphate-buffered saline. the supernatant was discarded, and bacterial pellet was then suspended in 200 µl of saline. 20 µL of Proteinase K solution was then added to resuspend bacteria and 200 µL of DNA lysis buffer was added and all were mixed by vortex and then incubated at 65 ℃ for 30 min. 200 µL of absolute ethanol was then added, mixed, and then all the mixture was transferred to spin column. The spin column was centrifuged at 10.000 × g for 1 min. 500 µL of washing buffer was added to the spin column and centrifuged at 10.000 × g for 1 min. The flow-through was discarded. This step was repeated, and the spin column was centrifuged at 13.000 × g for 2 min. The spin column was placed into a new 1.5 ml microfuge tube, 50 µL elution buffer was added. centrifugation at 13.000 × g for 1 min was done. The DNA was ready for further analysis. The concentration and purity of chromosomal DNA was determined using nanodrop (NanoDrop Technologies, Wilmington, DE, USA). The genomic DNA samples were stored at –70 °C before use.

### Oligonucleotide primers and PCR procedure

The sequences of the Oligonucleotide Primers used for the identification of *Mutans-Streptococci* species in this study are listed below^40^.

**Table:** 

Species	Primer	Sequence
*S. mutans*	GTFB-F	5ʹ -ACTACACTTTCGGGTGGCTTGG
	GTFB-R	5ʹ -CAGTATAAGCGCCAGTTTCATC
*S. sobrinus*	GTFI-F	5ʹ -GATAACTACCTGACAGCTGACT
	GTFI-R	5ʹ -AAGCTGCCTTAAGGTAATCACT

Reference: ^40^.

For PCR, primers GTFB-F and GTFB-R were designed to amplify a 517-bp DNA fragment of the *gtf* B sequence of *S. mutans*. and primers GTFI-F and GTFI-R were designed to amplify a 712-bp DNA fragment of the *gtf* I sequence of *S. sobrinus*. The PCR reaction mixture was prepared in a total volume of 25 μl including 5 μl of template DNA, 12.5 ul of 2X ABT Red master mix (Applied Biotechnology Co. Ltd, Egypt), and 20 Pico-moles of both forward and reverse primers. The volume was completed with nuclease-free water up to 25 μl. Reaction mixtures without a DNA template served as negative controls.

PCR proceeded in a thermal cycler (Perkin Elmer, Calif., USA) for 30 cycles. An initial denaturation step was done at 94 °C for 3 min then each cycle consisted of denaturation at 94 °C for 2 min, annealing at 51 °C for 1 min and extension at 72 °C for 2 min, with final extension at 72 °C for 5 min. PCR products were checked by electrophoresis on a 2.0% agarose gel in 1 X Tris–Borate-EDTA (TBE) buffer and staining with ethidium bromide. The images of the results were captured with a digital imaging system (Gene Genius, Bioimaging Sygene).

### Nano particles preparation and testing

#### Preparation of neem oil loaded PLGA biodegradable nanoparticles

The solvent displacement method was used to prepare the neem oil-loaded NPs. 7.5 mg of the drug (neem oil, UP Nature) was added to 6 ml of a solution containing acetone and 60 mg of the co-polymer Resomer® RG 502 H Poly (D, L-lactide-co-glycolide, Sigma-Aldrich, Germany) (PLGA). This solution was placed in an ultrasonic bath (H S walsh) to dissolve, forming the organic phase. Consequently, under moderate stirring (DIAHAN Scientific, Korea), the organic phase was added dropwise into 60 ml of an aqueous solution of sterile water (Milli-Q water) contain 600 mg of surfactant Kolliphor P188 (BASF, Germany) which constitutes the aqueous phase. After that, the organic solvents were evaporated by a rotaevaporation (heidolph, Germany) procedure using a rotavap 120 rpm at 40˚C for 20 min., the NPs were concentrated to a volume of 60 ml. 600 mg of carboxymethylcellulose (CMC) as a gelling agent was added to the formula to form the gel.

#### Preparation of Ca (OH)_2_ loaded PLGA biodegradable nanoparticles

The solvent displacement method was used to prepare Ca (OH)_2_-loaded NPs. 60 mg of the co-polymer Resomer® RG 502 H Poly (D, L-lactide-co-glycolide, Sigma-Aldrich, Germany) (PLGA) was added to 6 ml of acetone solution (Sigma Aldrich, Germany). This solution was placed in an ultrasonic bath (HS walsh) to dissolve, forming the organic phase. Consequently, under moderate stirring (DIAHAN Scientific, Korea), the organic phase was added dropwise into 60 ml of an aqueous solution of sterile water (Milli-Q water) contain 600 mg of surfactant Kolliphor P188 (BASF, Germany) and 7.5 mg of calcium hydroxide (Ca (OH)_2_) (JK dental vision) which constitutes the aqueous phase. After that, the organic solvents were evaporated by a rotaevaporation (heidolph, Germany) procedure using a rotavap 120 rpm at 40 ˚C for 20 min., the NPs were concentrated to a volume of 60 ml. 600 mg of carboxymethylcellulose (CMC) as agelling agent was added to the formula to form the gel.

#### Sample preparation for measurement of particle size and zeta potential

Samples were diluted 10X with deionized water then measured freshly after preparation.

#### Measurement of particle size and zeta potential

The prepared particles were analyzed for their particle size and size distribution including the average volume diameters and polydispersity index by photon correlation spectroscopy using particle size analyzer Dynamic Light Scattering (DLS) (Zetasizer Nano ZN, Malvern Panalytical Ltd, United Kingdom) at fixed angle of 173° at 25° C. Samples were analyzed in triplicate. The same equipment was used to determine zeta potential.

## Evaluation of antimicrobial efficacy

Efficacy of neem oil, neem NPs, calcium hydroxide NPs and conventional calcium hydroxide treatments were evaluated and compared for each of the isolated *Streptococcus* Species including *Streptococcus mutans, Streptococcus sobrinus,* and *Enterococcus faecalis* using disc diffusion method.

Different concentrations for each test substance were added to sterile discs and tested. Standard disc diffusion method was performed to test all clinically isolated strains for each tested substance according to CLSI standard protocols. Briefly, 4–5 similar overnight fresh colonies of each clinical isolate grown on TSA agar were used to make a bacterial suspension adjusted to a turbidity of 0.5 McFarland standard which corresponds to a density of 1.5 × 10⁸ cells /ml. The suspension was inoculated on Mueller Hinton agar plates. Sterile antibiogram discs (Oxoid, Hamptshire, UK) were impregnated with different concentrations of neem oil, neem NPs, calcium hydroxide, and calcium hydroxide NPs and applied to cultured Mueller Hinton agar plates and incubated. After 20 h of incubation, the diameters of the inhibition zones were read and compared. Clear areas of inhibition around each disc revealed the presence of antimicrobial activity for each compound used. For each concentration, 3 repeated determinations were performed.

## Evaluation of anti-biofilm effect

Antibiofilm effect was assessed by quantitative determination of biofilm forming capacity and biofilm inhibitory capacity of each tested compound against each of the isolated strains using Microtitre dish biofilm formation assay and an improved crystal violet assay method to reduce water loss from the peripheral wells and reducing the edge effect^41,42^.

Each of isolated bacterial strains was inoculated in Brain Heart Infusion broth (BHI) at 37 °C and 5% CO2. After 24 h incubation, growth was diluted 1: 100 and 10^6^ CFU/ml of *S. mutans* strains were inoculated with BHI broth and 2% sucrose in a 96-well microplate. Different concentrations of tested compounds were then added to the 96-well microplate. The plate was then incubated for biofilm formation for 24 h.

Crystal violet staining was used to estimate the biofilms biomass after biofilm formation. After fixing the biofilms in 96-well plates with methanol for 15 min, the supernatant was discarded, and the biofilms were air-dried. Then, biofilm was heat fixed by exposing the plates to hot air at 60 ℃ for 60 min. Then, 200 μl of 0.1% crystal violet was added into each well and incubated for 20 min. After the crystal violet was aspirated, the biofilms were cleaned with sterile deionized water, then air-dried. For quantitative analysis, the crystal violet stain in the biofilm was dissolved in 33% acetic acid, and its optical density (OD) was measured using Agilent BioTek ELx808 Absorbance Microplate Reader instrument at an absorbance of 590 nm.

Biofilm mass formed under each of the tested treatments was calculated according to the formula.

$$\frac{{Absorbance~of~treated~sample}}{{Absorbance~of~positive~control~sample}} \times 100$$

And the percentage of biofilm mass inhibition under each treatment was then calculated.

To determine the degree of biofilm formation, cut-off value (ODc) is first calculated. It is defined as three standard deviations (SD) above the mean OD of the negative control: ODc = average OD of negative control + (3 × SD of negative control). Then, the average OD value for each strain is calculated. For classification of the tested isolates according to their biofilm formation capabilities, the following parameters are used^43^ : OD ≤ ODc = no biofilm producer; ODc < OD ≤ 2 × ODc = weak biofilm producer; 2 × ODc < OD ≤ 4 × ODc = moderate biofilm producer;4 × ODc < OD = strong biofilm producer.

## In-silico testing of the interaction of neem components with *Streptococcus mutants * surface proteins

The 3D structure of 5 different ligands that could possibly act as active drug components was downloaded from PubChem database at https://pubchem.ncbi.nlm.nih.gov/. Target compounds tested included Nimbin (PubChem CID: 108058), Gedunin (PubChem CID: 12004512), Quercetin (PubChem CID: 5280343), Sitosterol (PubChem CID: 222284) and beta-d-Mannofuranoside, O-geranyl (PubChem CID:5365843).

Ten target proteins macromolecules that could function as surface proteins or adhesins from *Streptococcus mutans* were downloaded from PDB (Protein Data bank) database. Target proteins were prepared by removal of water, adding polar hydrogens, kollman charge and removal of ligands, cofactors, ions or other molecular structures using Autodock 4 and MGLtools version 1.5.7.^44,45^

 Molecular Docking of each of the tested ligands with all 10 target proteins was performed using Autodock. Analysis and visualization of output results was performed to find which interaction had the highest RMSD (Root Mean Square Deviation), higher binding affinity and conditions of hydrogen bonds was performed using PyMOL (The PyMOL Molecular Graphics System, Version 3.0 Schrödinger, LLC) and protein ligand profiler^46^.

### Statistical analysis

All tests were conducted in triplicate. Shapiro- Wilk test was carried out to test for normality of data distribution. Data were reported as means ± standard deviation (SD) for normally distributed variables and as median and IQR for non-normally distributed variables.

Statistical analysis for significance was determined using a two-tailed t-test assuming unequal variances with α = 0.5 and a P value ≤ 0.05 was considered as significant. Comparison of degree of antimicrobial inhibition (zone diameters) and biofilm mass reduction among the same isolated strains at different concentrations and treatments tested was performed using Friedman test. Post-hoc testing was performed using Wilcoxon signed rank test for pairwise intergroup comparisons to compare measured antibacterial and antibiofilm effect for the same tested strains under different tested conditions using SPSS Statistical package for analysis (SPSS® sofware, IBM).

## Supplementary Information

Supplementary Information.

## Data Availability

All data generated and analyzed in this work are presented in manuscript and its associated supplementary information.
