# Supplementary material for: In-vitro and In-silico evaluation of antimicrobial and antibiofilm effect of Neem oil and Calcium hydroxide nanoparticles against Mutans Streptococci and Enterococcus faecalis isolated from endodontic infections
Source: Sci Rep. 2024 Nov 2;14:26441. doi: 10.1038/s41598-024-75669-7 (PMC11531576; doi:10.1038/s41598-024-75669-7)
Supplement: Supplementary file 1 — Supplementary Information. [file 41598_2024_75669_MOESM1_ESM.pdf]

In-vitro and In-silico evaluation of antimicrobial and antibiofilm effect of Neem oil and Calcium hydroxide nanoparticles against *Mutans Streptococci* and *Enterococcus faecalis* isolated from endodontic infections.

Wedad M. Nageeb[1]*, Sherouk Hussein Adam[2], Nasr Hashem[3], Nelly Abdelsalam[4]

[1]Department of Medical Microbiology and Immunology, Faculty of Medicine, Suez Canal University, Ismailia, Egypt, wedad_saleh@med.suez.edu.eg

[2]Department of Endodontics, Faculty of Dentistry, Suez Canal University, Ismailia, Egypt, Shrouk_hussein@dent.suez.edu.eg

[3]Department of Endodontics, Faculty of Dentistry, Suez Canal University, Ismailia, Egypt, nasrrashad@yahoo.com

[4]Department of Endodontics, Faculty of Dentistry, Suez Canal University, Ismailia, Egypt

nellyendo@gmail.com

***Correspondence: Wedad M. Nageeb,** Department of Medical Microbiology and Immunology, Faculty of Medicine, Suez Canal University, Ismailia, Egypt

E-mail: wedad_saleh@med.suez.edu.eg

Supplementary Figure 1: Graphs showing Size Distribution Report by Intensity (A) and Zeta potential (B) for the three replicates measured for Neem oil NPs

S1 (A)

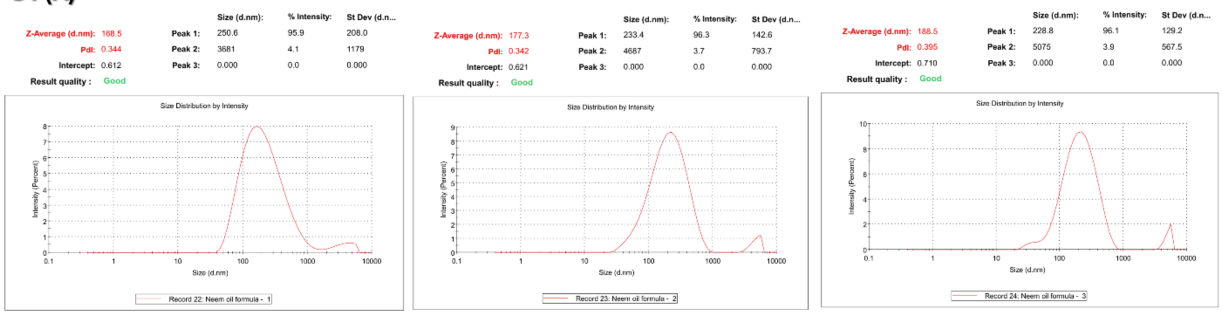

S1 (B)

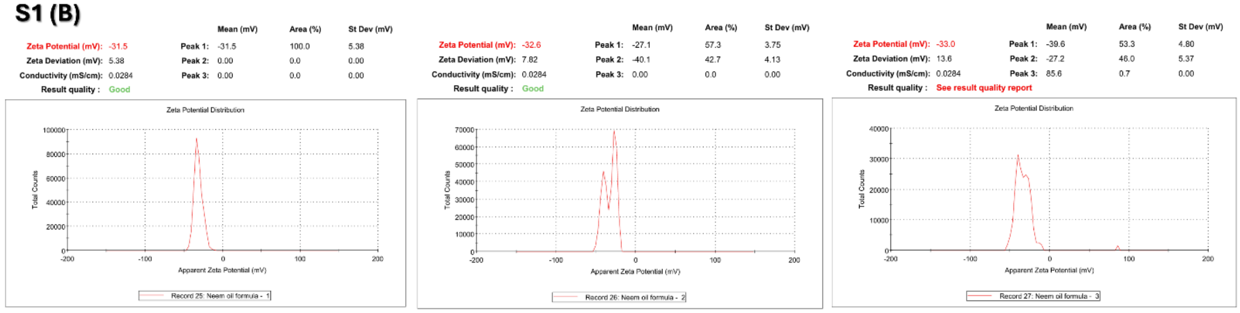

Supplementary Figure 2: Graphs showing Size Distribution Report by Intensity (A) and Zeta potential (B) for the three replicates measured for Ca $(OH)_2$ NPs

S2 (A)

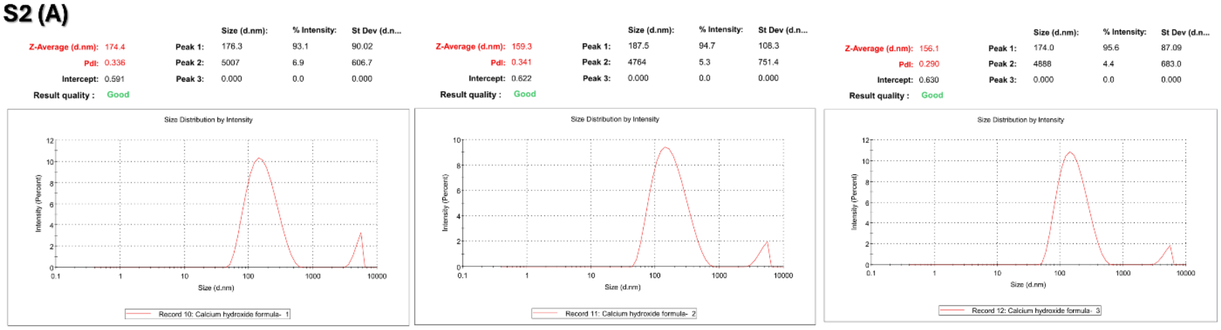

S2 (B)

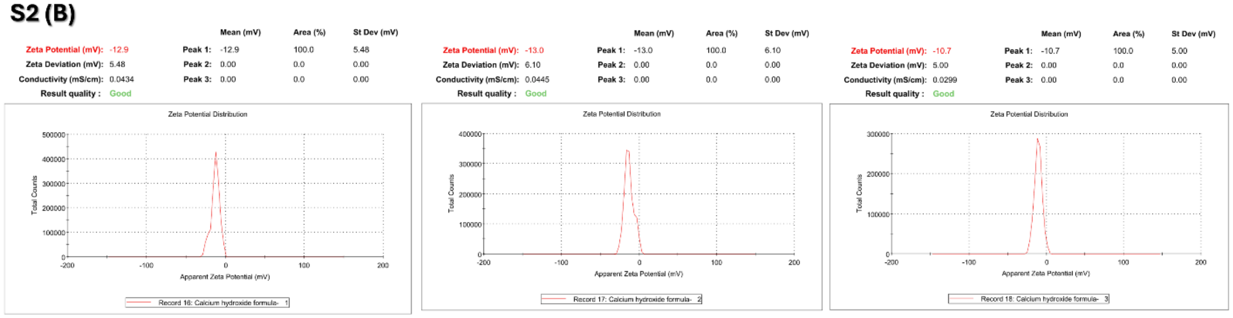

Supplementary Figure 3: Disc Diffusion antimicrobial susceptibility showing inhibition zones for tested compounds, **S3.A.** Inhibition Zones for Neem oil and Neem NPs preparations

S3 (A)

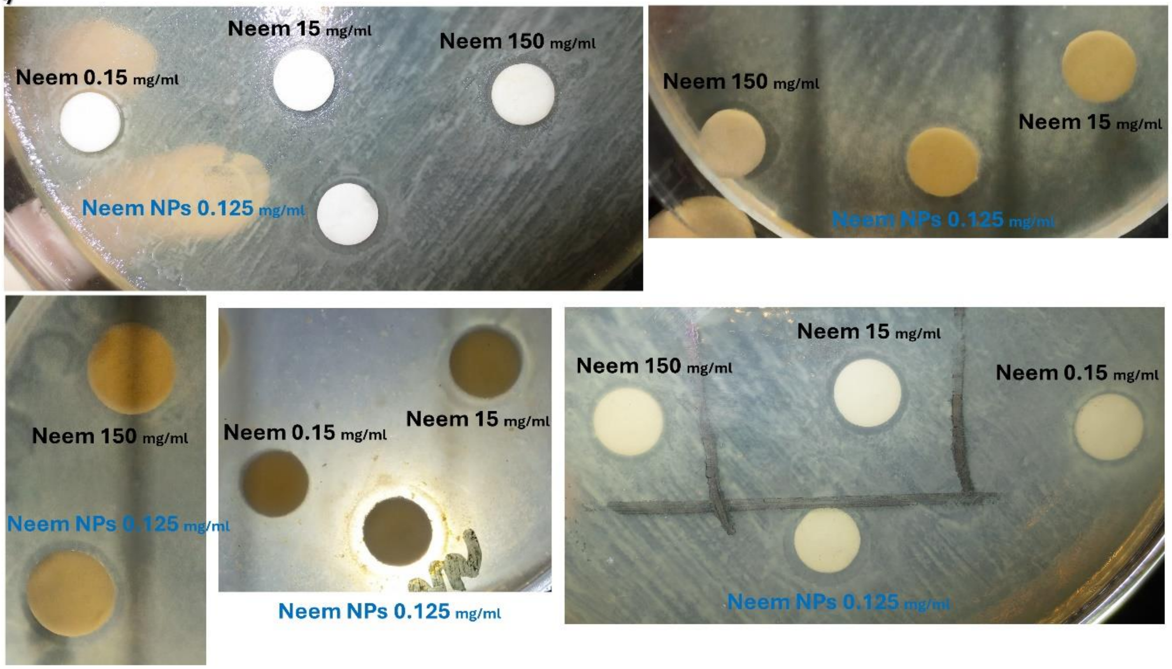

Supplementary Figure 3: Disc Diffusion antimicrobial susceptibility showing inhibition zones for tested compounds, **S3.B.** Inhibition Zones for Ca $(OH)_2$ and Ca $(OH)_2$ NPs preparations

S3 (B)

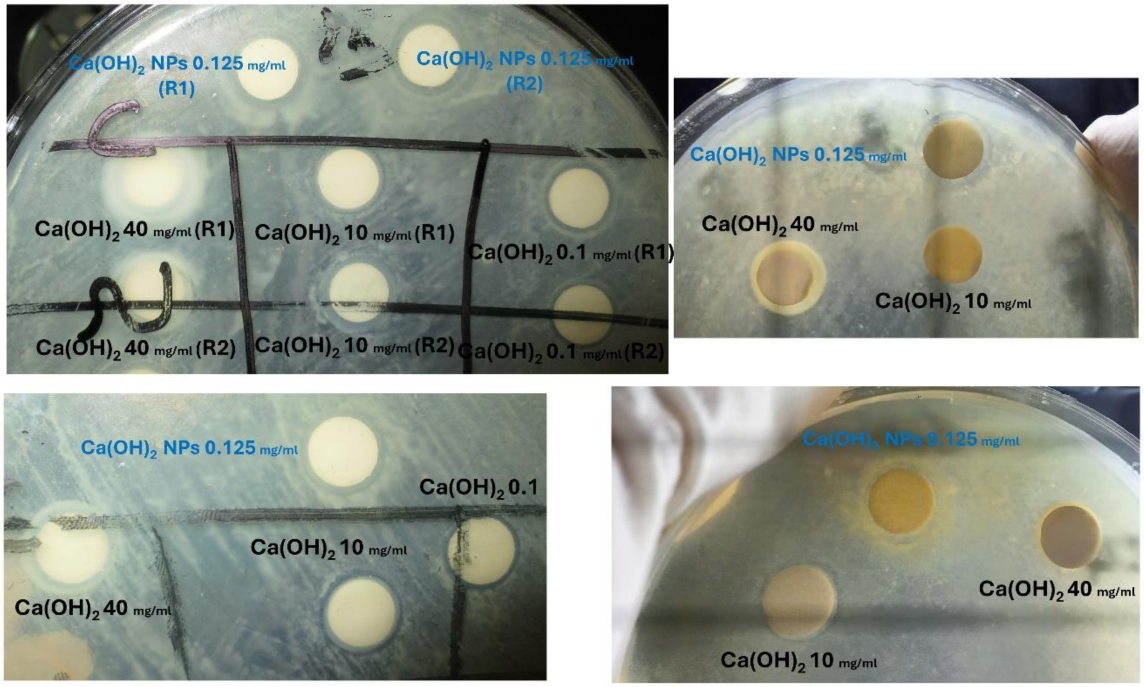

Supplementary Figures 4: The best docking poses and interaction bonds for the highest binding affinity observed of the tested neem active components.

S4.A. *Streptococcus mutans* GtfB, Glucosyltransferase-I (8fkl) interaction with Sitosterol

Streptococcus mutans GtfB, Glucosyltransferase-I (8fkl)
interaction with Sitosterol

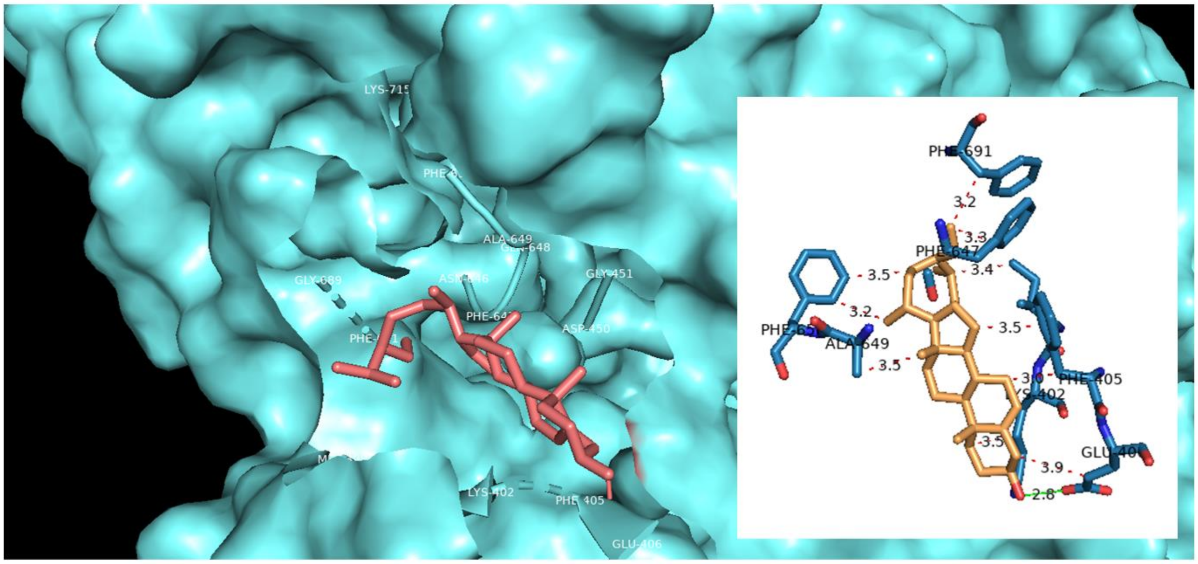

S4.B. *Streptococcus mutans* antigen I/II (AgI/II) cell surface-localized protein adhesin (3iox) interaction with Sitosterol

Streptococcus mutans antigen I/II (AgI/II) cell surface-localized protein adhesin (3iox)
interaction with Sitosterol

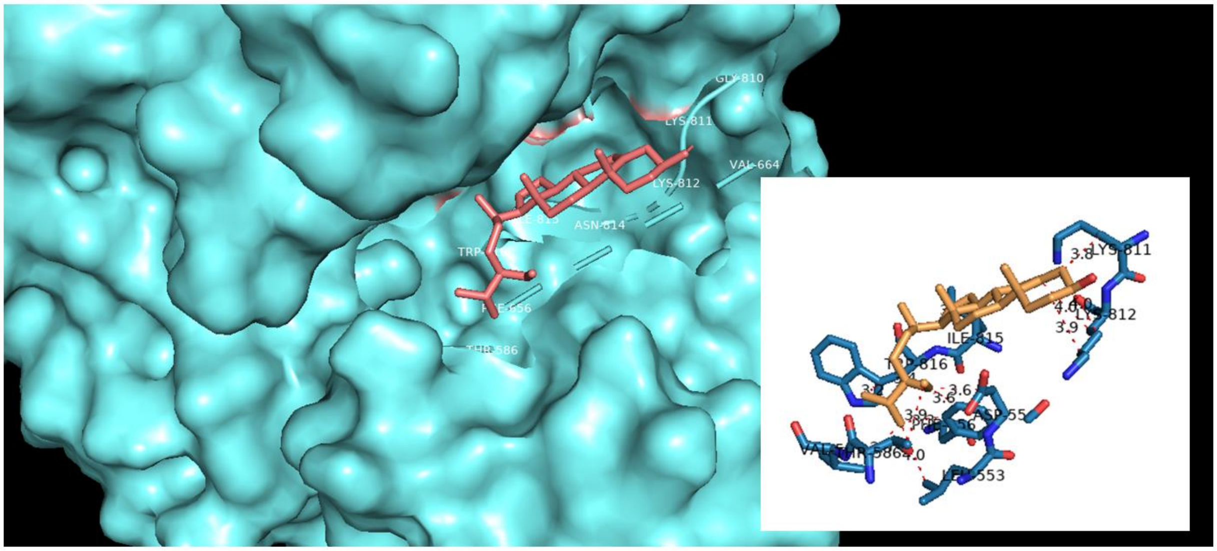

S4.C. *Streptococcus mutans* antigen I/II (AgI/II) cell surface-localized protein adhesin (3iox) interaction with Gedunin

Streptococcus mutans antigen I/II (AgI/II) cell surface-localized protein adhesin (3iox) interaction with Gedunin

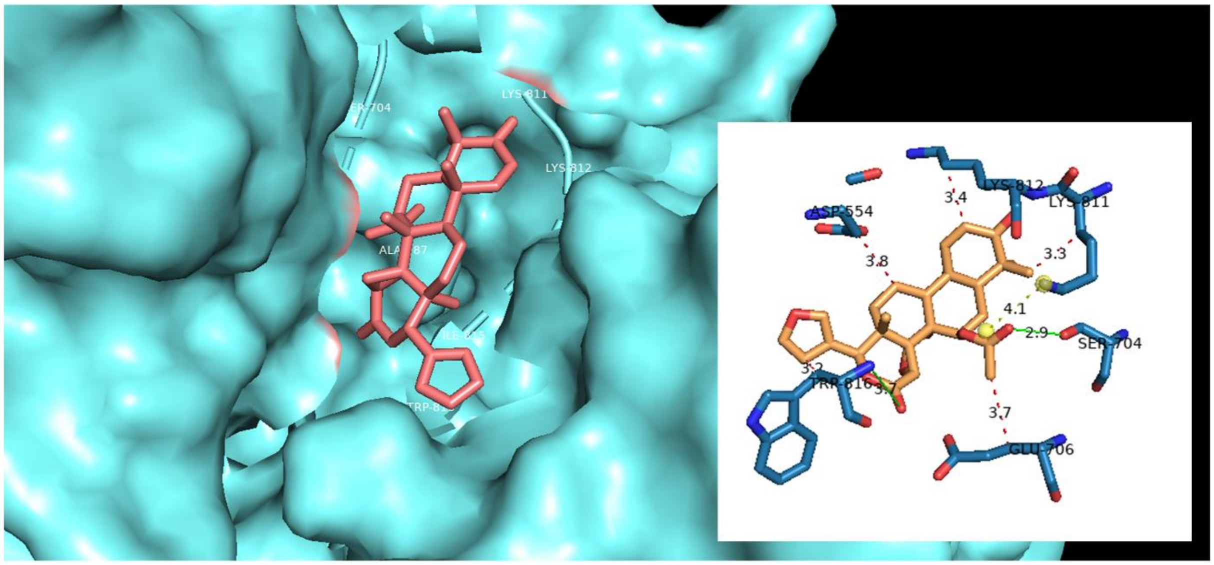

S4.D. *Streptococcus mutans* antigen I/II (AgI/II) cell surface-localized protein adhesin (3iox) interaction with Nimbin

Streptococcus mutans antigen I/II (AgI/II) cell surface-localized protein adhesin (3iox) interaction with Nimbin

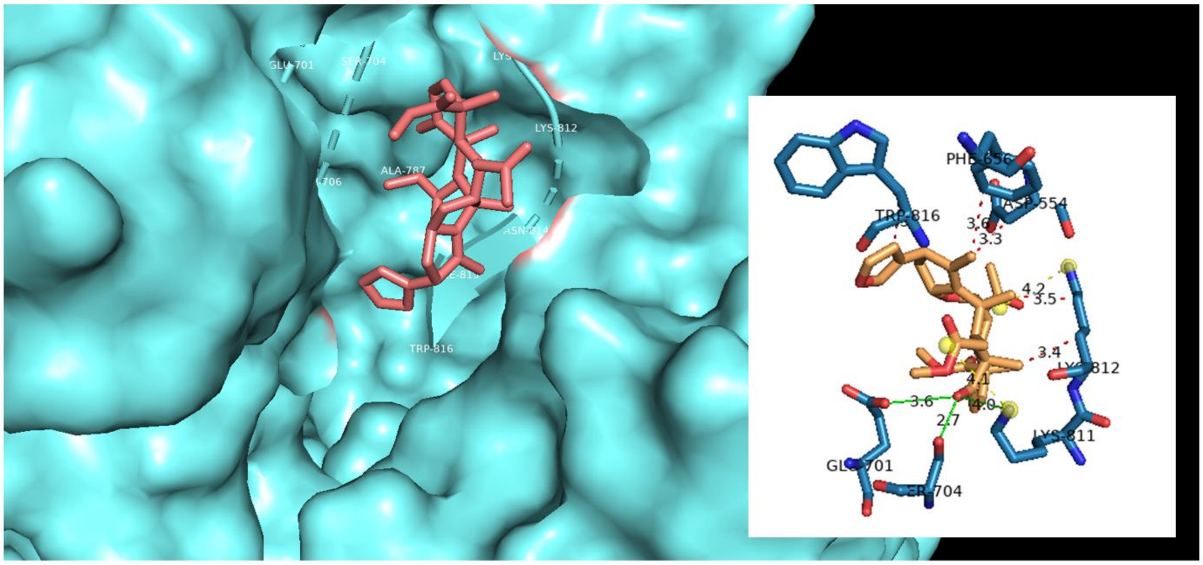

S4.E. Glucan Binding Protein C of *Streptococcus mutans* (5uqz) interaction with Quercetin

Glucan Binding Protein C of *Streptococcus mutans* (5uqz)
interaction with Quercetin

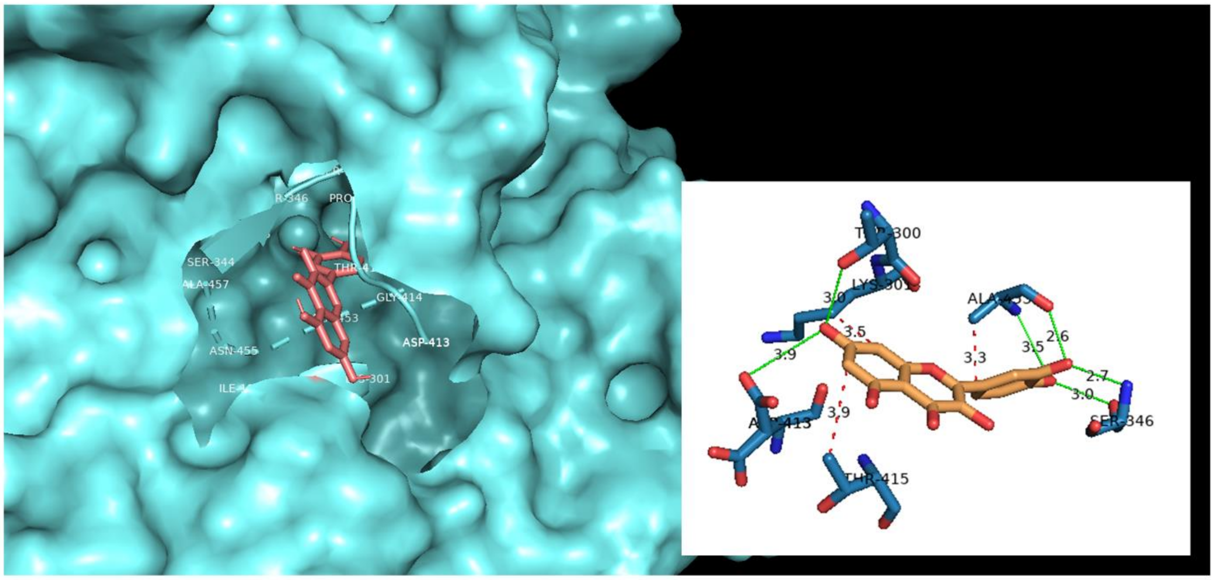

S4.F. *Streptococcus mutans* GtfB, Glucosyltransferase-I (8fkl) interaction with beta-d-Mannofuranoside, O-geranyl

Streptococcus mutans GtfB, Glucosyltransferase-I (8fkl)
interaction with beta-d-Mannofuranoside, O-geranyl

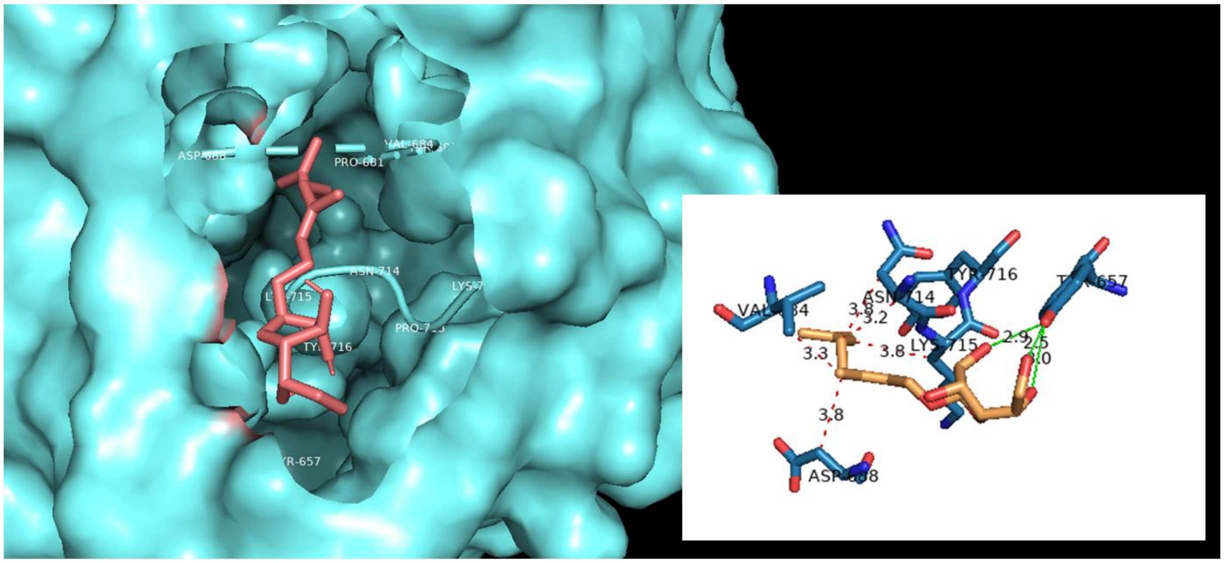

Supplementary Figures 5A: Full-Length PCR Gel Plot For Figure 2

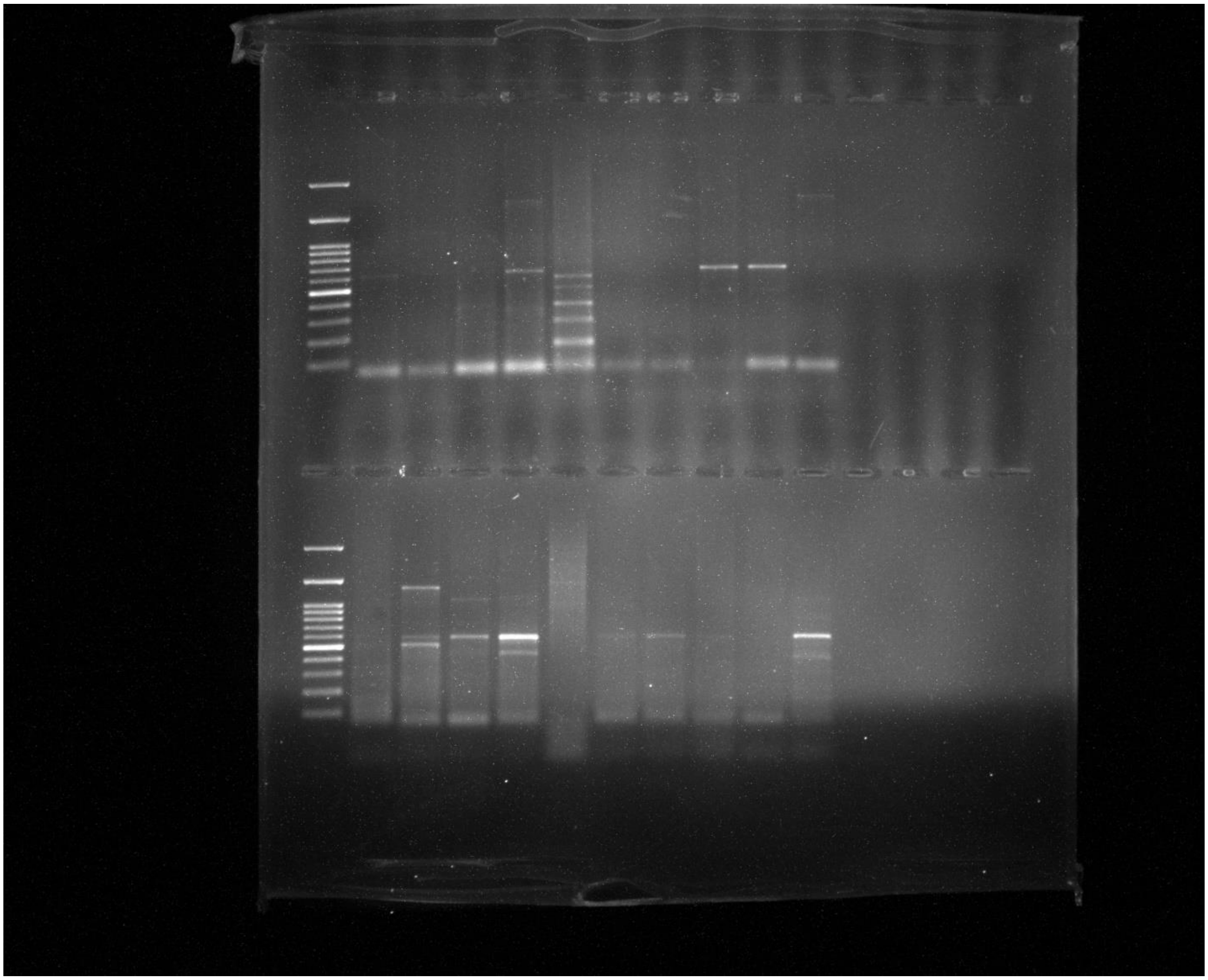

Figure 2 (A) Lower Part, Figure 2 (B) Upper Part, Refer to Figure 2

Supplementary Figures 5B: Full-Length PCR Gel Plot For Figure 2

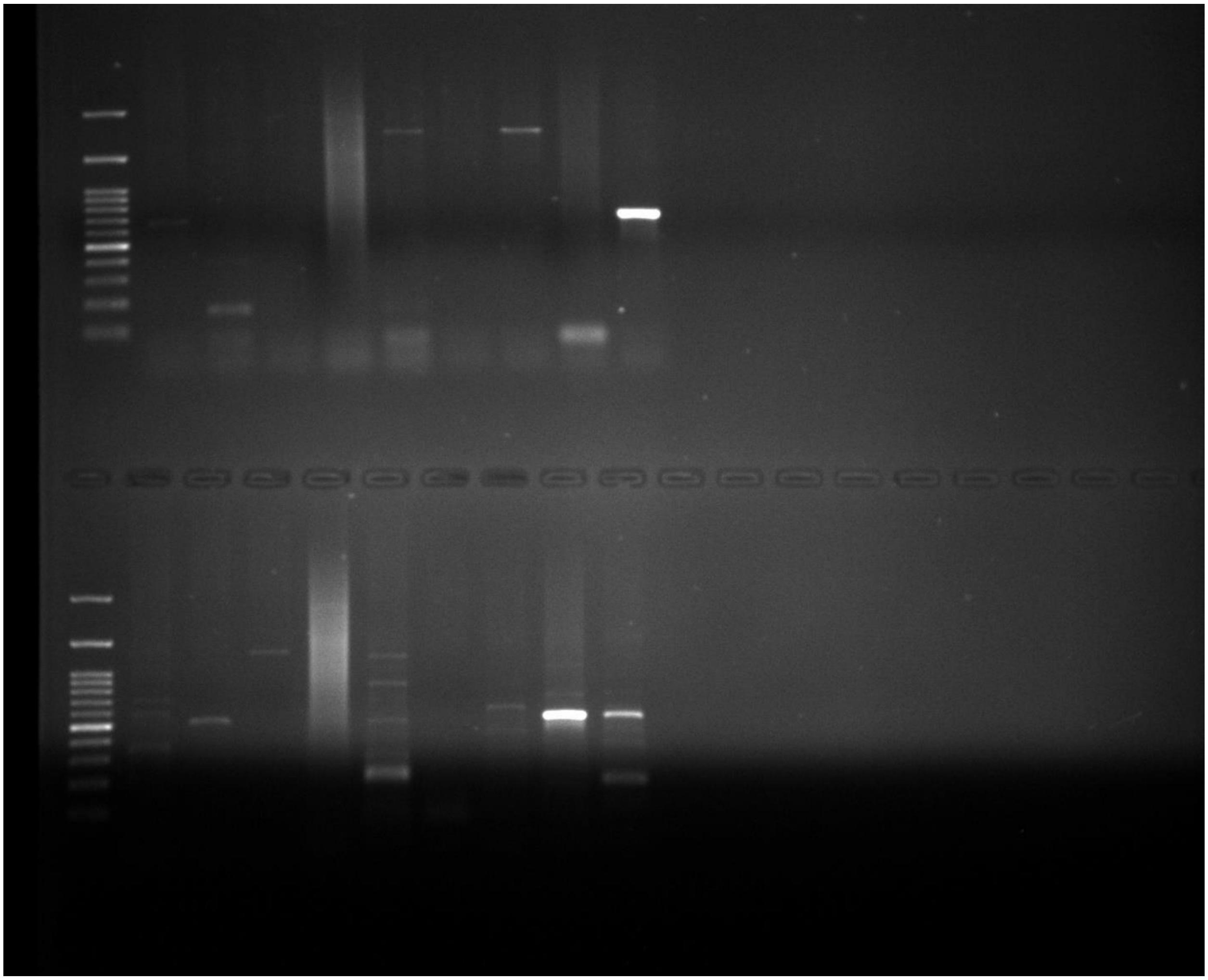

Figure 2 (A) Lower Part, Figure 2 (B) Upper Part, Refer to Figure 2
